# Dose-dependent induction of astrocyte activation and reactive astrogliosis in mouse brain following maternal exposure to carbon black nanoparticle

**DOI:** 10.1186/s12989-017-0184-6

**Published:** 2017-02-02

**Authors:** Atsuto Onoda, Ken Takeda, Masakazu Umezawa

**Affiliations:** 1 Department of Hygienic Chemistry, Graduate School of Pharmaceutical Sciences, Tokyo University of Science, 2641 Yamazaki, Noda, Chiba 278-8510 Japan; 2 The Center for Environmental Health Science for the Next Generation, Research Institute for Science and Technology, Organization for Research Advancement, Tokyo University of Science, 2641 Yamazaki, Noda, Chiba 278-8510 Japan; 3 Research Fellow of Japan Society for the Promotion of Science, 5-3-1 Kouji-machi, Chiyoda-ku, Tokyo, 102-0083 Japan; 4 Department of Materials Science and Technology, Faculty of Industrial Science and Technology, Tokyo University of Science, 6-3-1 Niijuku, Katsushika, Tokyo, 125-8585 Japan

**Keywords:** Nanoparticle, Carbon black, Maternal exposure, Brain, Astrocyte, Glial fibrillary acidic protein, Aquaporin-4, Western blotting, Immunohistochemistry, Immunofluorescence, Microarray

## Abstract

**Background:**

Recent studies indicate that maternal exposure to ambient ultrafine particles and nanoparticles has adverse effects of on the central nervous system. Quantitative dose–response data is required to better understand the developmental neurotoxicity of nanoparticles. The present study investigated dose-dependent effects of maternal exposure to carbon black nanoparticle (CB-NP) on astrocyte in the brains of mouse offspring.

**Methods:**

A CB-NP suspension (2.9, 15, or 73 μg/kg) was intranasally administered to pregnant ICR mice on gestational days 5 and 9. Cerebral cortex samples were collected from 6-week-old offspring and examined by Western blotting, immunostaining, microarray analysis, and quantitative reverse transcriptase-polymerase chain reaction. Placentae were collected from pregnant dams on gestational day 13 and examined by microarray analysis.

**Results:**

Maternal exposure to CB-NP induced a dose-dependent increase in glial fibrillary acidic protein (GFAP) expression in the cerebral cortex; this increase was particularly observed in astrocytic end-feet attached to denatured perivascular macrophages. Moreover, maternal CB-NP exposure dose-dependently increased aquaporin-4 expression in the brain parenchyma region around blood vessels. The changes in the expression profiles of GFAP and Aqp4 in offspring after maternal CB-NP exposure were similar to those observed in mice of a more advanced age. The expression levels of mRNAs associated with angiogenesis, cell migration, proliferation, chemotaxis, and growth factor production were also altered in the cerebral cortex of offspring after maternal CB-NP exposure. Differentially expressed genes in placental tissues after CB-NP exposure did not populate any specific gene ontology category.

**Conclusions:**

Maternal CB-NP exposure induced long-term activation of astrocytes resulting in reactive astrogliosis in the brains of young mice. Our observations suggest a potentially increased risk of the onset of age-related neurodegenerative diseases by maternal NP exposure. In this study, we report for the first time a quantitative dose–response relationship between maternal NP exposure and phenotypic changes in the central nervous system of the offspring. Moreover, our findings indicate that cortical GFAP and Aqp4 are useful biomarkers that can be employed in further studies aiming to elucidate the underlying mechanism of nanoparticle-mediated developmental neurotoxicity.

## Background

The development of nanotechnology provides several benefits to the global market, but is also accompanied by new potential health risks through occupational and environmental nanoparticles (NPs) exposure [1]. The safe handling and use of NPs first requires an understanding of the mechanism underlying NP exposure toxicity. Developmental and reproductive studies are widely recognized as important parts of toxicological science. In particular, various industrial chemicals are known to directly and indirectly affect the central nervous system during vulnerable stages of development [2, 3]. Clinical cohort and animal studies have revealed that prenatal exposure to particulate air pollution including black carbon is associated with an increased risk of brain developmental disorders such as autism spectrum disorder and schizophrenia in offspring [4–6]. However, information about the toxic effects of maternal NP exposure in developmental and reproductive toxicity studies remains limited [7–9]. In particular, few experimental animal studies have investigated the effects of prenatal exposure to carbon-based NPs on the developing central nervous systems of offspring [7, 8]. Therefore, the goal of the present study was to provide information on the developmental neurotoxicity of maternal exposure to carbon black nanoparticle (CB-NP).

Quantitative dose–response data is required for understanding developmental neurotoxicity. The US Environmental Protection Agency has indicated that dose–response data could help to reduce critical gaps in the current understanding of NP-associated developmental neurotoxicity [10]. However, only one in vivo study has reported the neurodevelopmental effects of NPs using multiple doses, and no dose–response relationship was observed in that study [11]. Additional quantitative dose–response studies evaluating maternal exposure to NPs are necessary to facilitate the risk assessment and hazard identification of NPs.

Previous studies have shown that maternal CB-NP exposure had adverse effects on the liver and brain of the offspring [11, 12]. While these studies have provided interesting insights, the sensitive target regions, affected cell types, and biomolecules in the brain remained to be elucidated. Our previous study demonstrated that maternal CB-NP exposure induced long-lasting diffuse perivascular abnormalities including histopathological changes in perivascular macrophages, the up-regulation of glial fibrillary acidic protein (GFAP) in astrocytes, and swollen astrocytic end-feet in the cerebral cortex [13]. The swelling of astrocytic end-feet related to maternal CB-NP exposure may be caused by changes in water transportation and ion homeostasis, which are notably regulated by aquaporin-4 (Aqp4) [14]. Hence, GFAP and Aqp4 may serve as quantitative and sensitive endpoints for the investigation of dose-dependent developmental toxicity after NP exposure, and increase knowledge on the mechanisms underlying subsequent neurobehavioral changes. Therefore, the present study investigated dose-dependent and long-term effects of maternal CB-NP exposure on astrocyte in the cerebral cortex of offspring mice. We used Western blotting to provide information on the dose-dependency of effects on GFAP and Aqp4 protein expression in astrocytes, immunostaining to elucidate the localization of these proteins in the cerebral cortex, and microarray as well as quantitative reverse transcription-polymerase chain reaction (qRT-PCR) analyses to examine comprehensive changes in gene expression in the placenta and brain of offspring after maternal CB-NP exposure.

## Methods

### CB-NP preparation

Printex 90 NP (Degussa Ltd., Frankfurt, Germany) were used for the CB-NP suspension. According to the manufacturer, the average primary particle size of Printex 90 NP is 14 nm, with a specific surface area of approximately 300 m^2^/g and an organic impurity content of less than 1% [12].

Printex 90 NP were suspended at a concentration of 2 mg/mL in ultra-pure water (10 mL), sonicated for 20 min using an ultrasonic cleaner, and immediately incubated on ice for 20 min. The intermediate phase (4 mL) was subjected to centrifugation at 16,000 × g for 20 min at 4 °C to remove bulk agglomeration. Finally, 2 mL of the supernatant suspension was collected and administered to mice in the high-dose (73 μg/kg) group. The suspension was diluted 5-fold and 25-fold with ultra-pure water for administration to the middle-dose (15 μg/kg) and low-dose (2.9 μg/kg) groups, respectively.

The size of secondary CB-NP in suspensions was characterized by transmission electron microscope (TEM; JEM 1200EXII, JEOL Ltd., Akishima, Tokyo, Japan) (irradiation current, 56 μA; acceleration voltage, 80 kV) on collodion-coated 200 Cu mesh (cat no. 6511, Nisshin EM Co. Ltd., Tokyo, Japan) and dynamic light scattering measurements using a NANO-ZS (Sysmex Co., Hyogo, Japan).

To determine the concentration of CB-NP in the each suspension, suspensions for administrations and standard suspensions (31.3, 62.5, 125 μg/mL) were concentrated from 1000 μL to 20 μL with a heating vaccum concentrator (MV-100, Tomy Seiko Co. Ltd., Tokyo, Japan), and 1 μL of each sample was dropped onto molybdenum single-hole sheet mesh (cat no. 09–1035, 0.3 mm, Okenshoji Co. Ltd., Tokyo, Japan), air-dried, and subjected to energy-dispersive X-ray spectrometry (EDX) under field emission-scanning electron microscope (FE-SEM; JSM-6500 F, JEOL Ltd., Tokyo, Japan) (accelerating voltage, 15 kV; magnification, 90×). CB-NP concentrations were calculated from the reduction of peak areas of molybdenum signal (2.290 keV) masked by carbon particles.

### Animals and treatments

**Fig. 1:**
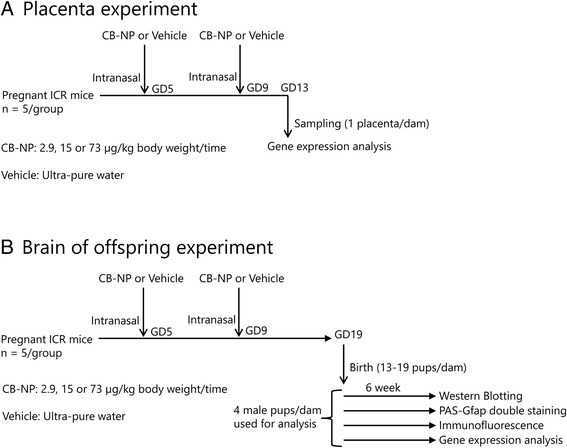
Summarized scheme of animal treatments and sample collection

Forty pregnant ICR mice (11 weeks of age) that were free of pathogens were purchased from SLC Inc. (Shizuoka, Japan), and housed separately in standard plastic cages. The animals were randomly allocated to one of four groups (*n* = 10/group; a control group, a low-dose group, a middle-dose group, and a high-dose group; Fig. 1a,b) and housed under pathogen-limited conditions with controlled temperature (22–24 °C) and relative humidity (50–60%) on a 12-h light/dark cycle with food and water available ad libitum. Animals were then randomly assigned to brain and placenta experiments. Pregnant mice were anesthetized with halothane, laid ventral side-up, and intranasally administered 1 mL/kg of CB-NP suspension (2.9, 15, 73 μg/mL) or ultra-pure water (0 μg/mL) into both nostrils. The treatments were performed on gestational days 5 and 9, as murine fetuses are particularly sensitive to various foreign substances during early gestation compared to any other fetal period [15, 16]. Placentae were collected from pregnant mice (*n* = 5/group) on GD13 (Fig. 1a). On postnatal day 1, the number of pups per dam was adjusted randomly to 11 or 12. Four male offspring mice per dam were randomly selected after weaning at three weeks of age, and brains were collected from these offspring mice at 6 weeks after birth (Fig. 1b) in order to assign randomly to (1) protein expression analysis by western blotting, (2) double-staining for GFAP and periodic acid Schiff (PAS) staining, (3) double-immunofluorescence staining for GFAP and Aqp4, and (4) gene expression analysis by microarray and qRT-PCR (Fig. 1b; one pup per litter for each outcome).

All experiments were performed in accordance with Animal Research: Reporting in vivo Experimental Guidelines for the Care and Use of Laboratory Animals [17] and were approved by the Tokyo University of Science Institutional Animal Care and Use Committee. All tissue sampling was performed under anesthesia with sodium pentobarbital (70 mg/kg, intraperitoneal) and all efforts were made to minimize pain and suffering.

### Protein extraction

**Fig. 2:**
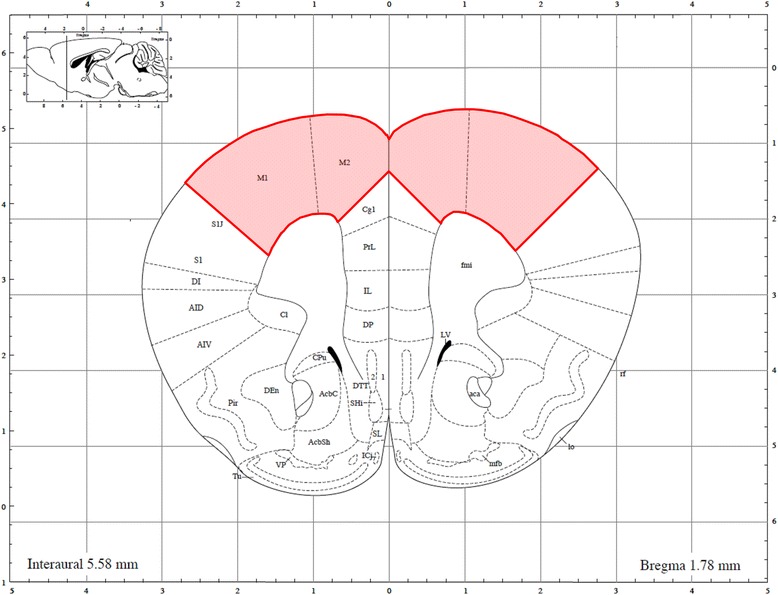
Collected/analyzed areas of the cerebral cortex in offspring mice (*red zone*)

Dissected cerebral cortices (Fig. 2) from 6-week-old male offspring (*n* = 5/group) were homogenized using the Biomasher II and Powermasher (Nippi Inc., Tokyo, Japan) in T-PER Tissue Protein Extraction Reagent (20 mL/g of tissue) (Takara Bio. Inc., Shiga, Japan) containing protease inhibitor cocktail (Complete tablet, EDTA-free, Roche Diagnostics, Basel, Switzerland) at 4 °C. Homogenates were centrifuged at 10,000 × g for 5 min at 4 °C to remove insoluble debris and then supernatants were collected for analysis. Supernatant total protein concentrations were determined by the bicinchoninic acid method using the Pierce BCA Protein Assay kit (Thermo Fisher Scientific K.K., MA, USA). Extracts were then stored at −80 °C until use.

### SDS-PAGE and western blotting

Protein extracts were mixed with denaturing sample buffer (125 mM Tris–HCl [pH 6.8], 20% glycerol, 4% w/v sodium dodecyl sulfate [SDS], 0.001% w/v bromophenol blue and 10% mercaptoethanol) and denatured by heating for 5 min at 95 °C. For each sample, 25 μg of protein per lane was loaded onto a 10% SDS-polyacrylamide gel, and electrophoresed initially at 110 V for 30 min at room temperature and subsequently at 150 V for 110 min at 4 °C. Separated proteins were electroblotted onto a polyvinylidene difluoride membrane (Merck Millipore, MA, USA) for 1 h at 400 mA at room temperature. After blocking with 5% skim milk in Tris-buffered saline (pH 7.4) containing 0.1% Tween-20 (TBS-T), membranes were incubated with primary rabbit polyclonal anti-GFAP antibody (code-no. Z0334, Dako Cytomation, Copenhagen, Denmark; 1:1000), rabbit monoclonal anti-β-actin (13E5) antibody (code-no. 4970, Cell Signaling Technology, Inc., MA, USA; 1:2000), or rabbit polyclonal anti-Aqp4 antibody (code-no. AB3594, Merck Millipore; 1:200) overnight at 4 °C followed by incubation with secondary horseradish peroxidase (HRP)-conjugated anti-rabbit IgG (code-no. sc-2004, Santa Cruz Biotechnology, Inc., Santa Cruz, CA, USA; 1:10000 for β-actin; 1:5000 for GFAP and Aqp4) for 1 h at room temperature. Between each step, immunoblots were washed thoroughly with TBS-T. Secondary antibody binding was visualized by chemiluminescence with Immobilon Westem Chemiluminescent HRP Substrate (Merck Millipore). To quantify detected signals, images were scanned using a ChemiDoc MP System (Bio-Rad Laboratories, Inc., TX, USA) and analyzed using Image Lab software (Bio-Rad Laboratories). Mouse GFAP molecules appeared at 55 kDa, corresponding to this molecule, on SDS-PAGE and also at 48 kDa (a GFAP-derived band). The densities of bands for GFAP at 55 kDa, β-actin at 46 kDa, and Aqp4 38 kDa (Aqp4) were quantified with background subtraction, and values for GFAP and Aqp4 were corrected to the corresponding value of β-actin in each sample.

### Double-staining for GFAP and periodic acid Schiff (PAS) staining-positive granules

Brains from 6-week-old male offspring mice (*n* = 5/group) were used for double-staining of GFAP and PAS-positive granules. Anesthetized mice were transcardially perfused with phosphate-buffered saline (PBS) and subsequently fixed by perfusion with 4% paraformaldehyde (PFA) in 0.1 M phosphate buffer. Brain samples were then post-fixed in 4% PFA in 0.1 M phosphate buffer for 24 h. Brains were embedded in paraffin and cut into 6-μm sagittal sections. Visualization of GFAP and PAS-positive granules was performed on paraffin sections using the appropriate antibodies and an avidin–biotin-peroxidase method. After blocking endogenous peroxidase by preincubation with 10% normal horse serum, sections were incubated in primary rabbit polyclonal anti-GFAP antibody (code-no. Z0334, DakoCytomation) diluted 1:1000 in PBS containing 0.1% Trion X (PBS-Tx) for 16 h at 4 °C. After rinsing 3 times for 5 min per rinse with PBS-Tx, sections were further incubated in secondary biotinylated donkey anti-rabbit IgG (code-no. AP182B, Chemicon, Temecula, CA, USA; 1:1000) for 120 min at room temperature and rinsed 3 times for 5 min per rinse with PBS-Tx. Sections were then treated with 1% periodic acid solution for 3 min, rinsed with distilled water for 1 min, and soaked in cold Schiff reagent for 60 min. Next, sections were soaked in sulfurous acid solution 3 times for 3 min per soak and then rinsed with distilled water for 1 min. Finally, sections were treated with an avidin-biotin-peroxidase complex (Vectastain ABC peroxidase kit, Vector Laboratories Inc., CA, USA; 1:400) for 120 min and reacted in a solution of 0.02% 3,3′-diaminobenzidine (DAB) in 0.1 M Tris–HCl buffer (pH 7.6) and 0.01% H_2_O_2_ for 20 min to detect peroxidase activity. GFAP immunoreactivity localized to the astrocytic cytoplasm was visible as light-brown staining. Sections were then washed in PBS, dehydrated in graded alcohol, cleared in xylene, and coverslips were applied with permount mounting medium (Thermo Fisher Scientific). Fifty sections (total 300 μm) from the longitudinal fissure of the cerebrum along sagittal plane were prepared from each mouse. One in every 5 sections was chosen (every 30 μm), and totally 10 sections per one mouse were subjected for PAS-GFAP staining analysis.

### Double-immunofluorescence staining for GFAP and Aqp4

Brains from 6-week-old (*n* = 5/group) male offspring mice and normally aged mice (as a positive control) were used for double-immunofluorescence staining of GFAP and Aqp4. Anesthetized mice were transcardially perfused with PBS and subsequently fixed with 4% PFA in 0.1 M phosphate buffer. Coronal sections (1 mm) of brains were post-fixed in 4% PFA in 0.1 M phosphate buffer for 5 h, cryoprotected in phosphate-buffered sucrose (10% sucrose, 4–6 h; 20% sucrose, 4–6 h; and 30% sucrose, 12–36 h) with 0.1% sodium azide, embedded in Tissue-Tek OCT compound (Sakura Finetek Japan Co., Ltd., Tokyo, Japan), frozen, and then cut into 10-μm sections.

Immunofluorescence was used to evaluate protein expression patterns of Aqp4 and GFAP in brain sections. Sections were blocked with 10% normal horse serum for 1 h at room temperature and then incubated with primary goat polyclonal anti-GFAP antibody (code-no. ab53554, Abcam, Cambridge, UK; 1:500) diluted 1:1000 in PBS for 16 h at 4 °C. After rinsing 3 times for 5 min per rinse with PBS, sections were further incubated with secondary Dylight 488-conjugated donkey anti-goat IgG (code-no. 605-741-125, Rockland Immunochemicals Inc., PA, USA; 1:1000) for 120 min at room temperature and rinsed 3 times for 5 min per rinse with PBS. Sections were further incubated with primary rabbit polyclonal anti-Aqp4 antibody (code-no. AB3594, Merck Millipore; 1:100) diluted 1:1000 in PBS for 16 h at 4 °C. After rinsing 3 times for 5 min per rinse with PBS, sections were then incubated with secondary Dylight 649-conjugated donkey anti-rabbit IgG (code-no. 611-743-127, Rockland Immunochemicals Inc; 1:1000) for 120 min at room temperature, rinsed 3 times for 5 min per rinse with PBS and twice for 5 min per rinse with distilled water, and nuclei were counter-stained with Hoechst 33342 (code-no. 346–07951, Dojindo Laboratories, Kumamoto, Japan). Thirty sections (total 300 μm) from the longitudinal fissure of the cerebrum along sagittal plane were prepared from each mouse. One in every 3 sections was chosen (every 30 μm), and totally 10 sections per one mouse were subjected for this immunofluorescence analysis.

### Line profiling of immunofluorescence in stained sections

Aqp4 expression in the cerebral cortex was further evaluated by fluorescence microscopy (Biorevo BZ-9000, Keyence Corporation, Osaka, Japan) of immunostained sections. Quantification was performed as per a previous study that detected changes in the expression level of GFAP and Aqp4 around blood vessels in the cerebral cortex [18]. The fluorescent intensity profile of Aqp4 was captured on a line that was set over a blood vessel 40-μm far from the vessel wall on either side into the surrounding brain tissue, and quantified with line profiling software (BZ-H2C, Dynamic Cell Count Vers.1.1, Keyence).

### Total RNA isolation

Dissected cerebral cortex and placenta tissues (*n* = 5/group) were homogenized in Isogen solution (Nippon Gene Co., Ltd., Tokyo, Japan). Total RNA was isolated with chloroform, purified with isopropanol, and precipitated in 70% ethanol according to manufacturer instructions, and finally dissolved in RNase-free water. RNA concentrations were determined by spectrophotometry at OD260 using a BioPhotometer Plus (Eppendorf, Hamburg, Germany). Extracted RNA from each sample was used for microarray and quantitative reverse transcription-polymerase chain reaction (qRT-PCR) analyses.

### Microarray analysis

After RNA purification by ethanol precipitation and using an RNeasy Micro Kit (Qiagen, Hilden, Germany), the integrity of extracted RNA was evaluated by capillary electrophoresis using a Bioanalyzer 2100 (Agilent Technologies, Inc., CA, USA). Total RNAs from 2–3 mice were pooled within each group. Each pooled RNA sample (*n* = 2/group) was labeled by Cy3 and hybridized to a SurePrint G3 Mouse GE 8x60K microarray (Agilent Technologies) according to a Takara Bio, Inc. protocol. The microarray was then washed using a Gene Expression Wash Buffers Pack (Agilent Technologies) and scanned with a DNA microarray scanner G2565CA (Agilent Technologies). Scanner output images were normalized and digitized using Agilent Feature Extraction software according to the Minimum Information About a Microarray Experiment (MIAME) guidelines [19] and a pre-processing method for Agilent data [20]. Expression threshold levels were set at > two-fold and < 0.5-fold in the high-dose group/control group comparison, with a correlation coefficient between nanoparticle concentration and gene expression of > 0.7 or < −0.7 in order to identify genes that exhibited dose-dependent changes in expression.

### Functional analysis of microarray data with gene ontology (GO)

To better understand the biological significance of the microarray results, a functional analysis was performed using gene annotation by GO. Genes were annotated with GO using an annotation file (gene2go.gz) provided by the National Center for Biotechnology Information (NCBI; MD, USA). The annotations used were last updated on June 20, 2015. Enrichment factors for each GO were defined as (nf/n)/(Nf/N), where nf is the number of flagged (differentially expressed) genes within a given category, Nf is the total number of genes within that same category, n is the number of flagged genes on the entire microarray, and N is the total number of genes on the microarray. GO with enrichment factors ≥ 2, nf ≥ 3 and *p* < 0.01 were extracted by Fisher’s exact test based on a hypergeometric distribution.

### qRT-PCR

Total RNA (1 μg) for each sample was used as a template to make the first strand of complementary DNA (cDNA) using M-MLV Reverse Transcriptase (Invitrogen Co., Carlsbad, CA, USA) according to manufacturer specifications. RT-PCR was performed using the SYBR Green Real-Time PCR Master Mix (Toyobo Co. Ltd., Osaka, Japan) and primers (Fasmac Co., Ltd. Kanagawa, Japan) or Real-Time PCR Master Mix (Toyobo) and TaqMan primer/probe sets (Applied Biosystems Japan, Tokyo, Japan) for the indicated genes. We selected 9 genes based on level of expression and plausible role in pathology from the GO analysis of microarray data. Primer and probe sequences are shown in Table 1. The values of target genes were normalized to the expression level of the housekeeping gene *GAPDH*.

**Table 1:** Primer and probe sequences for quantitative reverse transcription-polymerase chain reaction analyses

Gene			Sequence (5′–3′)	Tm (°C)
Gapdh	(NM_008084.2)	F	TGTGCAGTGCCAGCCTCGTC	60
		R	GGATGCATTGCTGACAATCT	
Sox17	(NM_011441)	F	AAGTAGCTCCAGAAACTGCAG	60
		R	CTGCTCATTGTATCCATGAGGTGA	
Tgfa	(NM_031199)	F	CTAGCGCTGGGTATCCTG	60
		R	CACTCACAGTGTTTGCGGAG	
Nos3	(NM_008713)	F	CTCTGCCTCACTCATGGGCACG	60
		R	GGATTTTGTAGCTCTTGTGCTGCTC	
Tbx1	(NM_001285472)	F	CTACCAGAATCACCGGATCACG	60
		R	CCGAGAGCGAGCAAAGGC	
		P	TAAGATTGCCAGCAACCCCTTCGCCAAAG	
Kdr	(NM_010612)	F	TCACCGGAAATCTGGAGAATCAG	60
		R	CTCAGTACAATGCCTGAATCTTCTAC	
		P	ACAACCATTGGCGAGACCATTGAAGTGACT	
Flt1	(NM_010228)	F	TGAGGAGCTTTCACCGAACTC	60
		R	ACTCGCTATTCTCAAGTCTATCTTCA	
		P	CTTGGTCTCAGTCCAGGTGAACCGCTTC	
Tie1	(NM_011587)	F	CCATGCTTTTGTCTACCAAAGTCA	60
		R	GCGGCATTCCCAGAACCC	
		P	AGCCAGACAGGACCACAGCAGAGTT	
Cyr61	(NM_010516)	F	ATCGCAATTGGAAAAGGCAGC	60
		R	GGCGTGCAGAGGGTTGAAA	
		P	AGGCTTCCTGTCTTTGGCACCGAACC	
Cxcl12	(NM_021704)	F	TCGCCAGAGCCAACGTCAA	60
		R	GATCCACTTTAATTTCGGGTCAATGC	
		P	CCAAACTGTGCCCTTCAGATTGTTGCACGG	

### Statistical analysis

All data are expressed as the mean ± SD. Numbers and sex ratios of pups at birth, offspring body weights at 6 weeks of age, protein expression levels, and mRNA expression levels were analyzed using a one-way ANOVA followed by Dunnett *post hoc* tests. Between-group differences in perivascular Aqp4-immunofluorescence were evaluated using unpaired t-tests. The level of significance was set at *p* < 0.05. Statistical analyses were carried out using Excel Statistics 2012 (Social Survey Research Information, Tokyo, Japan).

## Results

### Characterization of CB-NP suspensions

**Fig. 3:**
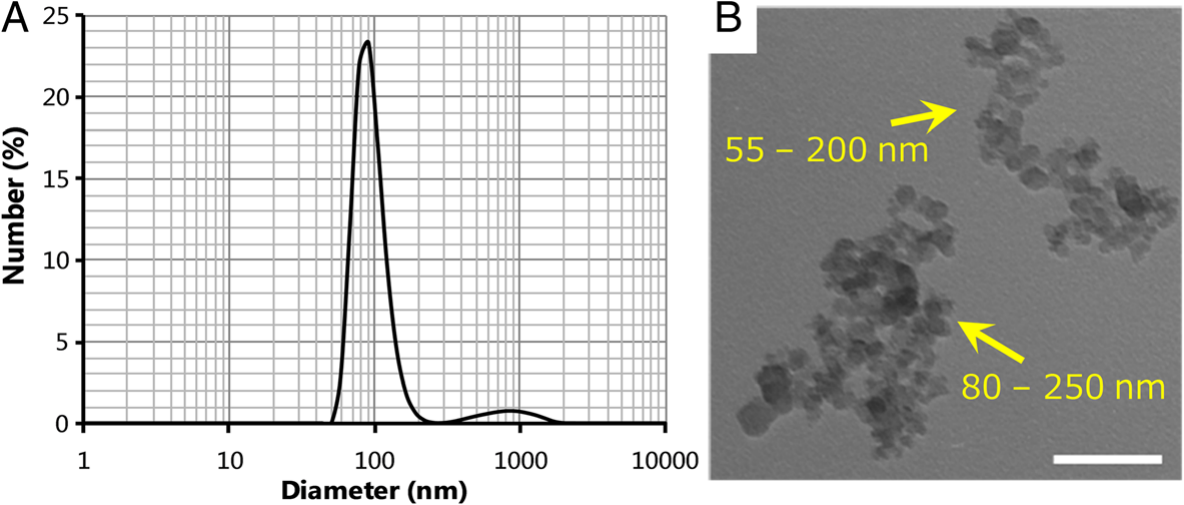
Characterization of carbon black nanoparticle (CB-NP) suspensions. **a** Transmission electron microscopy images of the high-dose CB-NP suspension. The *yellow* numerical value indicates the minor axis - major axis of secondary CB-NP particles. **b** Dynamic light scattering data of the high-dose CB-NP suspension after centrifugation. Scale bars represent 80 nm

DLS, TEM, and SEM/EDX were used to characterize CB-NP suspensions. DLS showed a major peak at 91.0 nm and a minor peak at 840 nm indicating the secondary diameter of CB-NP in administered suspensions (Fig. 3a). The 91.0-nm peak corresponded well with the typical size of small agglomerates of CB-NP observed under TEM. TEM analysis of the CB-NP suspensions showed that CB-NP consisted of open chain-agglomerates of 50–250 nm in diameter (Fig. 3b). The concentration of each CB-NP suspension for the high-dose group was 73 μg/mL, as shown by energy-dispersive X-ray intensity obtained using SEM/EDX; therefore the concentrations of 5- and 25-fold diluted suspensions were assumed to be 15 μg/mL and 2.9 μg/mL, respectively.

### Litter sizes and offspring body weights

There were no significant between-group differences in number or sex ratio of offspring litters at birth (Table 2) or offspring body weights at 6 weeks of age (Table 3).

**Table 2:** Effects of maternal exposure to carbon black nanoparticle on litter size and offspring sex ratio

Group name	Number of dams	Number of offspring	Sex ratio (%)^a^
Control (Ultra-pure water)	5	16 ± 2	47 ± 16
Low-dose CB-NP (2.9 μg/kg)	5	15 ± 2	44 ± 9
Middle dose CB-NP (15 μg/kg)	5	14 ± 2	42 ± 9
High dose CB-NP (73 μg/kg)	5	16 ± 2	53 ± 13

There were no significant between-group differences. Data are presented as the mean ± standard deviation. ^a^Sex ratio (%) = male/(male + female) × 100. *Abbreviations*: *CB-NP* carbon black nanoparticle

**Table 3:** Effect of maternal exposure to carbon black nanoparticle on male offspring body weight

Group name	Number of dams (Number of offspring)	Age	Body weight (g)
Control (Ultra-pure water)	5 (18)	6 weeks	33 ± 2
Low-dose CB-NP (2.9 μg/kg)	5 (18)	6 weeks	35 ± 3
Middle dose CB-NP (15 μg/kg)	5 (19)	6 weeks	33 ± 2
High dose CB-NP (73 μg/kg)	5 (20)	6 weeks	32 ± 1

There were no significant between-group differences. Data are presented as the mean ± standard deviation. *Abbreviations*: *CB-NP* carbon black nanoparticle

### GFAP expression levels in the cerebral cortex

**Fig. 4:**
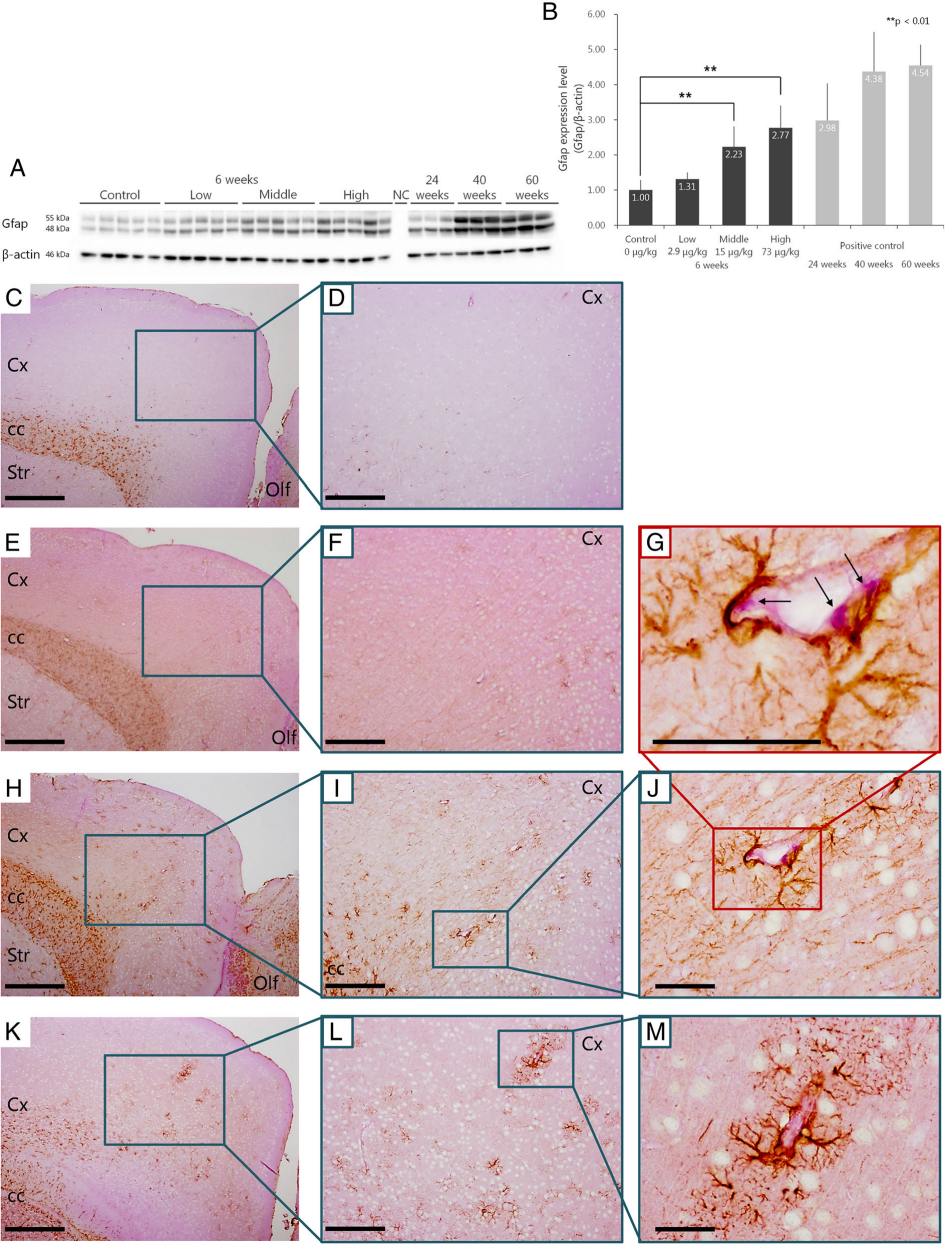
Expression level of glial fibrillary acidic protein (GFAP) in the cerebral cortices of offspring. **a**, **b** Quantification of GFAP protein expression in the cerebral cortices of offspring by Western blotting. A one-way ANOVA showed a significant effect of CB-NP treatment (F_3, 16_ = 17.46, *p* < 0.001). *Post hoc* Dunnett tests showed that GFAP protein expression was significantly (**p < 0.01) increased in the middle-dose and high-dose groups. **c**-**m**) Light micrographs of GFAP-positive astrocytes in the cerebral cortices of offspring. Scale bars represent 100 μm (**c**, **e**, **h**, **k**), 50 μm (**d**, **f**, **i**, **l**), and 10 μm (**g**, **j**, **m**), respectively. Cortices from 6-week-old male mice in the high-dose (**k**-**m**), middle-dose (**g**-**j**), and low-dose (**e**, **f**) groups (**e**, **f**) and control group (**c**, **d**) are shown. **d**, **f**, **i**, **l**) Enlarged views of panels **c**, **e**, **h**, and **k**. **j**, **m** Enlarged views of panels **i** and **l**. **g** Enlarged view of panel **j**. *Arrows* indicate enlarged PAS-positive granules. Data are presented as the mean ± standard deviation. Abbreviations: GFAP, glial fibrillary acidic protein; NC, negative control; Olf, olfactory bulb; Cx, cerebral cortex; cc, corpus callosum; Str, striatum

Western blotting was conducted to evaluate the expression level of GFAP in the cerebral cortices of offspring after maternal CB-NP exposure (Fig. 4a,b). A one-way ANOVA showed a significant effect of CB-NP maternal treatment (F_3, 16_ = 17.46, *p* < 0.001). *Post hoc* Dunnett tests showed that GFAP protein expression was significantly (**p < 0.01) increased in the middle-dose and high-dose groups relative to the control group (Fig. 4b). While GFAP expression in the cerebral cortex generally increases with age [21], GFAP expression in 6-week-old mice in the high-dose group was similar to that in mice of a more advanced age (24-week-old) (Fig. 4b).

Next, PAS-GFAP double staining was performed to identify the localization of GFAP. Few GFAP-positive astrocytes were observed in the cerebral cortices of mice from the control group (Fig. 4c,d) and those in the low-dose group (Fig. 4e,f). In contrast, a large number of GFAP-positive astrocytes was detected in the cerebral cortices of mice from the middle-dose and high-dose groups (Fig. 4g-m). In particular, GFAP expression was remarkable in areas surrounding blood vessels (Fig. 4j,m). GFAP-positive astrocytic end-feet were also observed attached to perivascular macrophages with enlarged PAS-positive granules (Fig. 4g).

### Aqp4 expression in the cerebral cortex

**Fig. 5:**
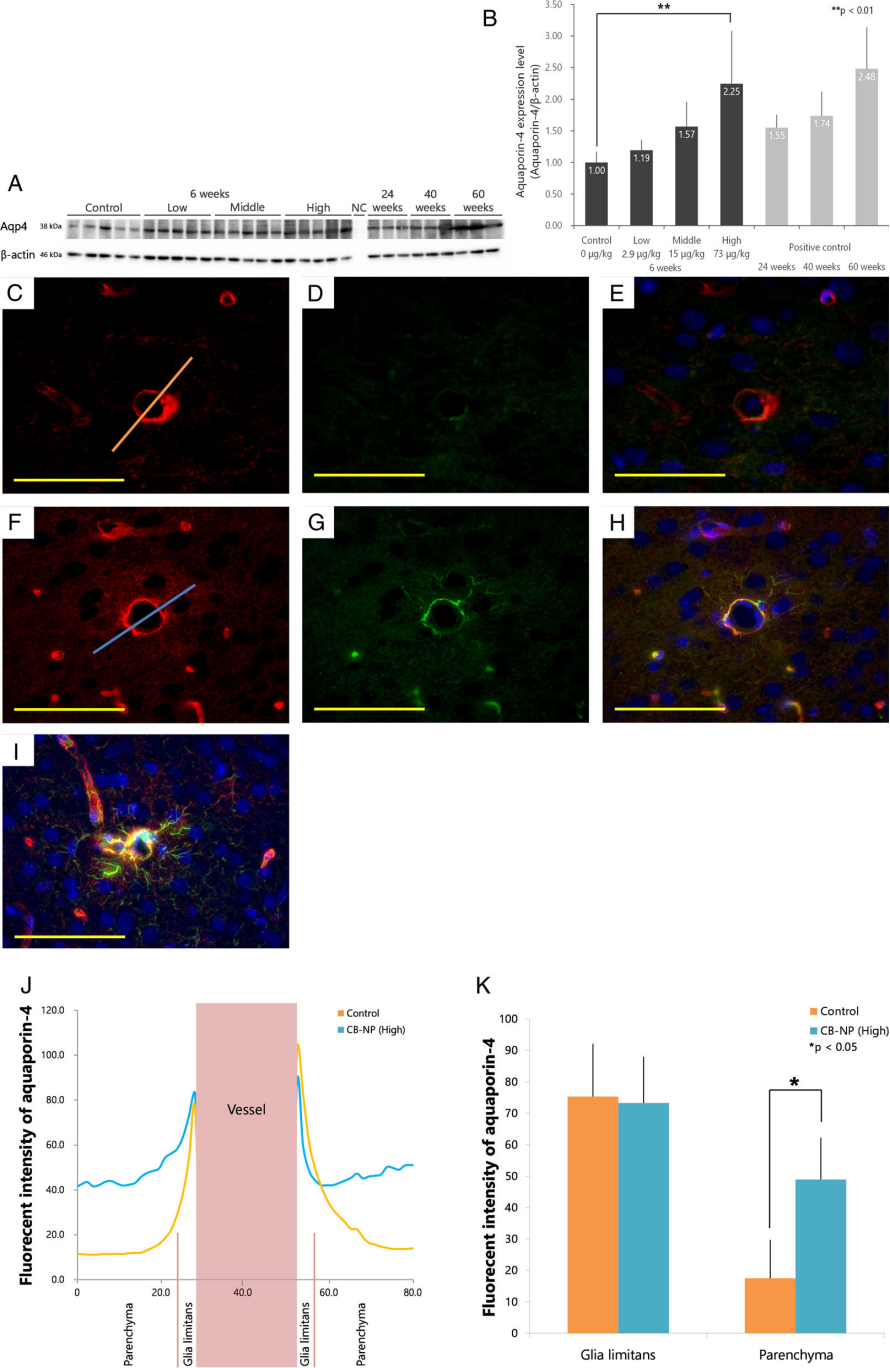
Expression level of aquaporin-4 (Aqp4) protein in the cerebral cortices of offspring. **a**, **b** Quantification of Aqp4 protein in the cerebral cortices of offspring by western blotting. A one-way ANOVA showed a significant effect of CB-NP treatment (F_3, 16_ = 6.95, *p* < 0.01). *Post hoc* Dunnett tests showed that Aqp4 protein expression was significantly (*p < 0.05) increased in the high-dose group. **c**-**k** Fluorescent micrographs of Aqp4 and glial fibrillary acidic protein (GFAP) expression in the cerebral cortices of 6-week-old offspring and mice of a more advanced age (60-week-old). All scale bars represent 80 μm. The cerebral cortices of 6-week-old male mice from the control (**c**-**e**) and high-dose (**f**-**h**) groups are shown. **i** Cerebral cortices of 60-week-old male mice as a positive control. **c**, **f** Aqp4; **d**, **g** GFAP; **e**, **h**, **i** merge of Aqp4 *(red*), GFAP (*green*), and nuclear stain (*blue*). **j**, **k** Quantification of Aqp4 immunofluorescence in linear regions placed over cerebral blood vessels (*orange line* on **c** and *light blue line* on **f**). *p < 0.05, unpaired *t*-test. Data are presented as the mean ± SD. Abbreviations: Aqp4, aquaporin-4; GFAP, glial fibrillary acidic protein; NC, negative control

Western blotting were carried out to investigate the expression levels of Aqp4 in the cerebral cortices of offspring after maternal CB-NP exposure (Fig. 5a,b) because Aqp4 is also one of the important molecules for the function of astrocytes and blood–brain barrier. Aqp4 expression was dose-dependently increased in the cerebral cortex in response to maternal CB-NP exposure, similar to the effect observed on GFAP expression. A one-way ANOVA showed a significant effect of CB-NP maternal treatment (F_3, 16_ = 6.95, *p* < 0.01). *Post hoc* Dunnett tests showed that Aqp4 protein expression was significantly (*p < 0.05) increased in the high-dose group relative to the control group (Fig. 5b). While Aqp4 expression in the cerebral cortex also generally increases with age [22], Aqp4 expression in 6-week-old mice from the high-dose group (73 μg/kg) was similar to that in mice of a more advanced age (60-week-old) (Fig. 5b).

The detailed localization of Aqp4 was evaluated by double-immunofluorescence staining (Aqp4 + GFAP) and line profiling of fluorescence intensity in immunostained brain sections. In the control group, Aqp4 immunofluorescence was localized proximal to blood vessels (glia limitans region) and otherwise low in brain tissue (parenchyma region > 5 μm from vessels) (Fig. 5c,j,k). In the high-dose group, Aqp4 expression was significantly (*p* < 0.05) increased in brain parenchyma region around blood vessels relative to the control group (Fig. 5f,j,k). GFAP expression was increased in astrocyte end-feet around blood vessel in the cerebral cortex of the high-dose group compared to the control cortex (Fig. 5d,g). Aqp4 expression was most notably increased in GFAP-positive astrocytes by maternal CB-NP exposure (Fig. 5h).

### Gene expression profiling by microarray and annotation analyses

RNAs from the cerebral cortex were subjected to a microarray analysis to comprehensively investigate differences in gene expression among the experimental groups. Of 62972 spots (28950 mRNAs) printed on the microarray, 30700 spots (22908 mRNAs) produced high-quality signal after incubation with cerebral cortex samples. From these 30700 spots, 1168 spots (1126 mRNAs) exhibited up-regulation > 2-fold or down-regulation < 0.5-fold in the high-dose group relative to the control group. From these 1168 spots, 278 spots (268 mRNAs) were flagged with a correlation coefficient (an index of dose-dependency) of > 0.7 or < −0.7. Functional analysis with GO revealed that the 268 flagged mRNAs were significantly enriched in GO terms related to blood vessels (angiogenesis, blood vessel patterning, positive regulation of endothelial cell proliferation, vasculogenesis, blood vessel development, and ventricular septum development), cell proliferation and growth factors (positive regulation of cell migration, positive regulation of mesenchymal cell proliferation, growth factor binding, and positive regulation of endothelial cell proliferation), and chemotaxis/positive regulation of cell migration (Table 4).

**Table 4:** Significantly enriched gene ontology categories according to cerebral cortex microarray data

Gene ontology	Enrichment factor	*P*-value	Gene symbol up-regulation	Gene symbol down-regulation
Angiogenesis	3.66	<0.001	*Flt1, Kdr, Nos3, Ptprb, Robo4, Sox17, Tbx1, Tgfa, Tie1*	
Positive regulation of mesenchymal cell proliferation	10.11	<0.001	*Foxf1, Foxp2, Kdr, Tbx1*	
positive regulation of epithelial cell proliferation	6.64	<0.001	*Erbb2, Foxp2, Kdr, Tbx1, Tgfa*	
Growth factor binding	9.5	<0.001	*Cyr61, Erbb2, Flt1, Kdr*	
Patterning of blood vessels	8.47	0.001	*Cxcl12, Flt1, Lef1, Tbx1*	
Ventricular septum development	14.7	0.001	*Cyr61, Luzp1, Stra6*	
Chemotaxis	4.56	0.005	*Ccr3, Cmtm2b, Cxcl12, Cyr61, Flt1*	
Positive regulation of endothelial cell proliferation	5.7	0.005	*Ccr3, Cxcl12, Kdr, Rptor*	
Vasculogenesis	5.7	0.005	*Foxf1, Kdr, Sox17, Tie1*	
Blood vessel development	5.41	0.006	*Foxf1, Stra6, Tbx1, Tie1*	
Positive regulation of cell-substrate adhesion	7.13	0.008	*Cyr61, Foxf1, Nid1*	
Positive regulation of cell migration	3.36	0.009	*Cxcl12, Cyr61, Kdr, Lef1, Flt1, Foxf1*	

Expression levels of 268 mRNAs were altered in a dose-dependent fashion in the high dose group versus the control group. Functional features of these 268 mRNAs were extracted using gene annotation by gene ontology (enrichment factor ≥ 2, *p* value ≤ 0.01, symbol number ≥ 3). Enrichment factors for each Gene ontology were defined as (nf/n)/(Nf/N), where nf is the number of flagged (differentially expressed) genes within a given category, Nf is the total number of genes within that same category, n is the number of flagged genes on the entire microarray, and N is the total number of genes on the microarray

mRNAs from placenta tissues were also subjected to microarray analysis. Of 62972 spots (28950 mRNAs) printed on the microarray, 31781 spots (18269 mRNAs) produced high-quality signal after incubation with placenta samples. From these 31781 spots (18269 mRNAs), 817 spots (417 mRNAs) exhibited up-regulation > 2-fold or down-regulation < 0.5-fold in the high-dose group relative to the control group. From these 817 spots, 26 spots (19 mRNAs) were flagged with a correlation coefficient of > 0.7 or < −0.7. Functional analysis with GO revealed that the flagged 19 mRNAs were not significantly enriched in any GO terms.

### qRT-PCR gene expression analysis of the cerebral cortex

**Fig. 6:**
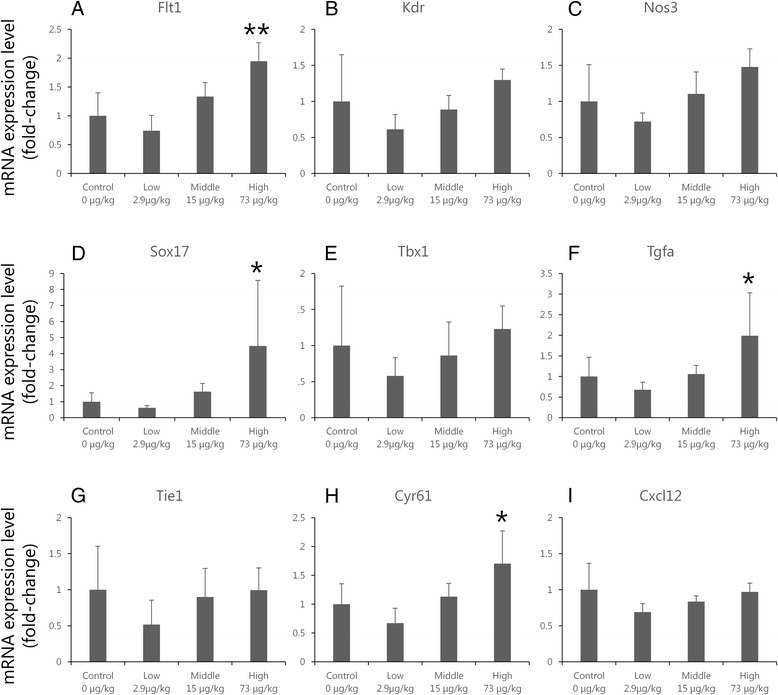
Expression levels of mRNA in the cerebral cortices of offspring. Expression levels of mRNA selected from microarray data and the gene annotation analysis were analyzed by quantitative reverse transcription-polymerase chain reaction. Relative expression levels of the target genes were calculated for each sample after normalization against *Gapdh*. A one-way ANOVA followed by *post hoc* Dunnett tests revealed significant increases in *Flt1* (**a**), *Sox17* (**d**), *Tgfa* (**f**), and *Cyr61* (**h**) gene expression in the cerebral cortices of high-dose group offspring compared to the control group. *p < 0.05, **p < 0.01 vs. control. *Kdr* (**b**), *Nos3* (**c**), and *Tbx1* (**e**) showed non-significant increases in gene expression in the cerebral cortices of high-dose group offspring compared to the control group. Up-regulation of Tie1 (**g**) and Cxcl12 (**i**) gene expression was not observed in each groups

RT-PCR was conducted in order to validate the microarray data and to obtain expression data for individual samples. mRNA expression levels of *Sox17*, *Tgfa*, *Flt1*, and *Cyr61* in the cerebral cortex were significantly increased in the high-dose group relative to the control group (*Sox17*, *Tgfa*, and *Cyr61*: *p* < 0.05 vs. control; *Flt1*: *p* < 0.01 vs. control). mRNA expression levels of *Nos3*, *Tbx1*, and *Kdr* in cortices from offspring tended to increase in a dose-dependent fashion following maternal exposure to CB-NP (Fig. 6), but these changes were non-significant.

## Discussion

Quantitative dose–response analyses are critical components of toxicological science [10]. However, no study to date has evaluated the dose–response relationship of developmental neurotoxicity in offspring after maternal exposure to NPs [7–9]. This literature deficit may be in part due to a lack of previously established biomolecules or behavioral parameters for use in quantitative dose–response analyses. In the present study, we found that maternal CB-NP exposure dose-dependently increase cortical GFAP expression in offspring mice, particularly in astrocytic end-feet attached to perivascular macrophages with PAS-positive enlarged lysosomal granules. Maternal CB-NP exposure also dose-dependently increased cortical Aqp4 expression in offspring mice, particularly in GFAP-positive astrocytes in the brain parenchyma region around blood vessel. This is therefore the first study to report a quantitative dose–response relationship between maternal exposure to NPs and phenotypic changes in the central nervous system of offspring mice. Moreover, our findings indicate that cortical GFAP and Aqp4 are useful quantitative endpoints for assaying NP-related developmental neurotoxicity.

The use of astrocytic GFAP and Aqp4 as toxicological endpoints also provide information on the molecular and cellular effects of NPs developmental neurotoxicity. Astrocytes in the cerebral cortex (protoplasmic astrocytes) extend end-feet to neighboring blood vessels to participate in the formation and maintenance of the blood–brain barrier [23]. In other words, astrocytes form a first line of brain defense by regulating blood–brain barrier function [24, 25]. GFAP is an important marker of astrocyte activation [26, 27] and reactive astrogliosis in response to brain injury [28–30], and astrogliosis facilitates the reconstruction of the blood–brain barrier and tissue remodeling local to sites of injury [31, 32]. Increases in astrocytic GFAP expression are frequently accompanied by increases in Aqp4 expression [33, 34]. Aqp4 is a water-selective channel that is highly expressed in the perivascular membrane of astrocytic end-feet in the central nervous system [14]. Accordingly, Aqp4 plays important roles in regulating the flow of brain fluids, ion homeostasis, and astrocyte plasticity in response to a variety of injuries [14]. Furthermore, previous studies have indicated that increases in Aqp4 expression are associated with blood brain barrier leakage, astrogliosis, and microglial activation in the context of brain injury [33, 34]. Taken together, increased GFAP and Aqp4 expression in the present study suggests that maternal CB-NP exposure may have induced chronic astrocyte activation, reactive gliosis, and blood brain barrier leakage in developing offspring.

Changes in the expression profiles of GFAP and Aqp4 in offspring after maternal CB-NP exposure were similar to those in observed in mice of a more advanced age. Many previous studies have shown that diffuse reactive astrogliosis is a general feature of the aging brain [18, 35, 36]. Indeed, age-related increases in astrocytic GFAP and Aqp4 expression have been observed in the central nervous system [21, 22]. Moreover, astrocyte activation and an increase in GFAP are also observed in age-related neurodegenerative disease such as dementia including Alzheimer’s disease [23]. The increase in Aqp4 expression level is observed in the brain parenchyma region in normally aged mice [18], whereas acute insults such as cerebral ischemia elevate Aqp4 expression level in the astrocytic-endfeet on glial limitans [37–39]. To this end, it is notable that alterations in Aqp4 regulate the exchange of cerebrospinal fluid and interstitial fluid (the glymphatic system) to affect the clearance of waste including amyloid β [40, 41], and that the age-related dysregulation of Aqp4 expression impairs this waste clearance function [18]. In the present study, maternal exposure to NPs had similar effects to that of aging and accordingly, it can be speculated that these changes might predispose offspring to age-related brain disorders. In agreement with this speculation, a recent cohort study of air pollution suggested that long-term exposure to ambient fine particles promoted the effects of aging in the brain [42]. Further investigations of the effects of NPs on astrocytes can improve our understanding of the effects, mechanisms, and repercussions of particulate neurotoxicity.

The present study also examined the effects of maternal CB-NP exposure on gene expression in the brains of offspring and in placentae. Comprehensive alterations in cortical mRNA expression were associated with angiogenesis, cell migration, proliferation, chemotaxis, and growth factor dysregulation in offspring. Another study of CB-NP toxicity using intratracheal instillation also show significant alterations of expression levels of mRNA related to chemotaxis, inflammation, and cell cycle in liver of offspring by comprehensive gene expression analysis [43]. In particular, Sox17, Tgfa, Flt1, and Cyr61 were dose-dependently increased by maternal CB-NP exposure and may be potential key molecules regulating the histopathological changes of perivascular regions induced by NP-associated neurotoxicity because these genes are related to regulation of the homeostasis and remodeling of the blood vessels in the brain [44–47]. Maternal inhalation of titanium dioxide NP also lead to significant fetal microvascular dysfunction [48]. The published evidence and the data from the present study indicate that the denaturation of vascular development in the fetus is important for understanding the developmental toxicity of the nanoparticles. Of note, astrocytes surrounding blood vessels play crucial roles in growth factor regulation, angiogenesis, cell migration, and proliferation [49, 50]. Thus, the dysregulation of these processes might be related to chronic perivascular damage induced by maternal CB-NP exposure in the brain. In particular, angiogenesis in the cerebral cortex is disrupted by excessive neural stimulation in the postnatal period [51]. It can be hypothesized that maternal exposure to CB-NP perturbed neural activity in developing offspring during the postnatal period; however, no direct evidence exists in support of this hypothesis. Further investigation is required to clarify the potential effects of maternal exposure to NPs on neuronal activity, and the contribution of these effects to behavioral and histopathological changes during the postnatal development of offspring.

Of note, while the present study demonstrated effects of maternal CB-NP exposure in the cerebral cortices of offspring mice, similar effects may have been diffusely distributed throughout various other brain regions. A previous study indicated that maternal airway exposure to CB-NP caused diffuse and subchronic perivascular abnormalities in the brains of offspring [13]; in particular, lysosomal granule enlargement and honeycomb-like ultrastructure were observed in perivascular macrophages, which play a part in blood–brain barrier function to protect the brain against circulating foreign matter and pathogen and to remove waste flowing from the central nervous system into the cerebrospinal fluid [52, 53], consistent with the present study. Diffuse histopathological abnormalities related to the maternal environment may be important for understanding the effects of maternal exposure on the risk of developing a brain disorder, and warrant further investigation in future studies.

In contrast to our above results, dysregulated genes in the placenta did not populate a specific GO term in our annotation analysis. Epidemiological studies have shown that maternal exposure to ambient fine particles such as PM_2.5_ and diesel exhaust particles is associated with fetal malnutrition and low birth weight [54, 55], potentially related to placental impairment [56–58]. In fact, maternal exposure to diluted diesel exhaust rich in NP induce disturbance of placental functions including placental vascularization [59]. In the present study, we did not find evidence for placental impairment, for abnormalities in offspring birth weights, or for functionally significant mRNA dysregulation in the placenta after maternal CB-NP exposure; this may have been due to the use of CB-NP at relatively low doses in our study (2.9, 15, and 73 μg/kg). Moreover, the placental response to external stimulus is actually different between male and female in fetus [60, 61]. Therefore, the analysis considering the difference of sexes will elucidate the effects of maternal CB-NP exposure on placenta in more detail.

The kinetics of NPs are important for understanding the mechanisms underlying the effects of NP exposure. NPs with diameters of 1–100 nm can traverse various biological barriers in mammals including the blood-air barrier [62–64] and the blood-placental barrier [65]. Moreover, NPs are transferred from the dam to the fetal brain and liver [66] and can be detected in the brains of offspring mice even after birth [67]. Therefore, it is possible that NPs directly led to adverse effects on development through maternal exposure in the present study; however, CB-NP may not translocate to the astrocytes surrounding blood vessels in the brain. Again, this may have been due to the relatively low doses of CB-NP used in our study. Indeed, a previous study also failed to detect CB-NP using transmission electron microscope in perivascular macrophages or astrocytes of offspring from mothers intranasally exposed to CB-NP (95 μg/kg) [13]. Therefore, it is also important to consider the indirect effects of NP exposure on the fetus such as inflammation and oxidative stress in the dam [12, 68], as these mechanisms may have also led to developmental neurotoxicity in the present study.

Finally, the exposure dose of CB-NP in the present study and in the real world merits discussion. The concentration of CB-NP workers in carbon factories are potentially exposed to has been estimated at up to 14 mg/m^3^ [69]. Predicted pulmonary deposition of CB-NP aerosolisation based on observed particle size distribution is estimated to be 35% [12]. Given that the daily respiratory volume of a woman of 50 kg is approximately 15 m^3^, the amount of CB-NP female workers are exposed to by inhalation is approximately 24 mg/8-h, assuming that 35% particle deposit in the respiratory organs. 24 mg CB-NP/50 kg/8 h/day corresponds to 480 μg CB-NP/kg/day. In the present study, the high-dose group was exposed to a concentration of 73 μg CB-NP/kg/day. Thus, the doses used in the present study are highly relevant for an occupational setting. In addition, the doses used in the present study are also lower than doses used in other studies of CB-NP toxicity using intratracheal instillation [43]. Although the dose rate of intranasal administration, an experimental model of pulmonary exposure in rodents, is temporarily higher than the dose rate of inhalation of an aerosol, we believe that our approach adds valuable insights to the field for two reasons: (1) The dose of CB-NP used in the present study is lower than the dose used for inhalation exposure in a previous study (42 mg/m^3^, 1 h/day, 11 days) [12] and (2) The dose of CB-NP employed in the current study approximates the estimated dose for workers in carbon factories. Nevertheless, further investigations of the effects of maternal inhalation of NP on astrocytes in the offspring brain are needed to allow for a comprehensive risk assessment and to further our understanding of the developmental neurotoxicity of maternal NP exposure.

## Conclusions

The present study showed dose-dependent and long-term induction of maternal CB-NP exposure on astrocyte activation and reactive astrogliosis in the cerebral cortices of offspring mice. The astrocyte activation by maternal CB-NP exposure was similar to those in observed in mice of a more advanced age. Astrocytic GFAP and Aqp4 expression may play key roles in the mechanisms underlying NP-related developmental neurotoxicity, and can accordingly serve as quantitative and sensitive endpoints for the prediction of NP-associated toxicities in future studies. Moreover, maternal CB-NP exposure dysregulated mRNAs associated with angiogenesis, cell migration, proliferation, chemotaxis, and growth factor production including *Sox17*, *Tgfa*, *Flt1*, and *Cyr61* in the brains of offspring mice. The present data will aid future investigations examining the features and mechanisms of NP-associated developmental neurotoxicity, and moreover contribute to the establishment of protective strategies against occupational and unintended NP exposure.

## Abbreviations

Aqp4: Aquaporin-4
CB-NP: Carbon black nanoparticle
cc: Corpus callosum
Cx: Cerebral cortex
DAB: 3,3′-diaminobenzidene
EDX: Energy-dispersive X-ray spectrometry
FE-SEM: Field emission-scanning electron microscope
GFAP: Glial fibrillary acidic protein
GO: Gene ontology
HRP: Horseradish peroxidase
MIAME: Minimum information about a microarray experiment
NC: Negative control
NP: Nanoparticle
Olf: Olfactory bulb
PAS: Periodic acid Schiff
PBS: Phosphate-buffered saline
PBS-Tx: Phosphate-buffered saline containing 0.1% trion X
PFA: Paraformaldehyde
qRT-PCR: Quantitative reverse transcription-polymerase chain reaction
SDS: Sodium dodecyl sulfate
SEM: Scanning electron microscope
Str: Striatum
TBS-T: Tris-buffered saline (pH 7.4) containing 0.1% tween-20
TEM: Transmission electron microscope
